# Epidemiological profile of mucosal melanoma in Brazil

**DOI:** 10.1038/s41598-019-57253-6

**Published:** 2020-01-16

**Authors:** Daniel Cohen Goldemberg, Andreia Cristina de Melo, Livia Cristina de Melo Pino, Luiz Claudio Santos Thuler

**Affiliations:** 1 National Cancer Institute of Brazil (INCA), Rio de Janeiro, Brazil; 2 Medical School of Valença (UNIFAA), Rio de Janeiro, Brazil; 3 Federal University of the State of Rio de Janeiro (UNIRIO), Rio de Janeiro, Brazil

**Keywords:** Cancer epidemiology, Metastasis

## Abstract

Mucosal melanomas are primary malignant neoplasias originated from melanocytes within mucous membranes in any part of mucosal surface lining, more commonly, in the nasal cavity and accessory sinuses, oral cavity, lips, pharynx, vulvar, vaginal, cervix and anorectal mucosa. Epidemiology data regarding mucosal melanomas in Brazil is scarce, hence the motivation to conduct this research paper. The χ2 test was used to compare categorical variables. Forward stepwise logistic regression method was used in the multivariate analysis to identify independent predictors of early death. A total of 801 patients were included in the analysis. Surgical resection is frequently the first approach to primary tumours (65.3%), even though the utility of lymph node surgery and radiation therapy is not well established. Advanced stage was observed in more than two thirds of patients. Early death was observed in 28.3%. MM cases with regional or distant metastases as well as those located in unusual locations had almost 4 times more risk for early death. Besides that, MM located in lips, oral cavity and pharynx and those receiving chemotherapy had 2 times more risk of early death.

## Introduction

Mucosal melanoma (MM) is a primary malignant neoplasia originated from melanocytes within mucous membranes in any part of mucosal surface lining, more commonly, the head and neck region, followed by anorectal mucosa and vulvovaginal mucosa^1–3^. MM is rare and represents approximately 1% of all melanoma cases^1^. On top of that, the disease has a terrible prognosis, with a five year survival rate of only 25% compared to 80% in cutaneous melanomas^1,2^.

Feller and collaborators state that regardless of the treatment approach, MM is constantly fatal^4^. Notwithstanding, Ascierto and collaborators believe that the advances in melanoma treatment, especially related to the new discoveries on the scope of the molecular profile of these tumours, boosted the optimism when it comes to the possibility of more effective systemic therapies available^5^.

There is a lack of nationwide studies regarding MMs in Brazil. The purpose of this research was to fill this gap clarifying the scientific community about the epidemiological characteristics of MM in the country with special emphasis on MM’s early death predictors.

## Results

Considering the Hospital Cancer Registry, 801 cases of MM were registered for sixteen years in Brazil (Table 1). Vulva, vagina and uterine cervix are the most prevalent sites with 33.7% of cases, followed by lips, oral cavity and pharynx (23.1%), anal and rectal (17.9%) and nasal and paranasal mucosae (17.7%).

**Table 1:** Anatomic location of Mucosal Melanoma in Brazil.

Anatomic location	N	%
Vulva, vagina, and uterine cervix*	270	33.7
Lips, oral cavity and pharynx	185	23.1
Anal and rectal	143	17.9
Nasal and paranasal	142	17.7
Other mucosae**	61	7.6
**Total**	**801**	**100**

*Vulva (n = 212), vagina (n = 43) and uterine cervix (n = 15).

**Other mucosae: digestive organs (n = 28), respiratory tract and intrathoracic organs (n = 12), penis and other male urinary tract non-specified MM (n = 12), genitourinary tract (n = 6), peritoneum and retroperitoneum (n = 3).

In men, from 2000 to 2016, the most frequent anatomic location of MM was labial, oral cavity and pharyngeal, with 93 (35.6%) out of 261 cases. On the other hand, women presented 270 (50.0%) cases related to vulva, vagina and cervix out of 540 cases. Patients present with MM usually at more advanced age, particularly from 50 to 79 years old (two thirds of cases). A total of 26.0% of patients present with MM from 60 to 69 years old, with the highest frequency for each subtype of MM, with the exception of vulva, vaginal and cervix, with 22.2%, although very close to 23.0% of patients ranging from 70–79 years old (Table 2).

**Table 2:** Demographic characteristics of Mucosal Melanoma in Brazil.

Variables	Anatomic location					Total	p-value
	Vulva, vagina and uterine cervix	Lips, oral cavity and pharynx	Anal and rectal	Nasal and paranasal	Other mucosae*		
Sex**							0.308
Male	—	93 (50.3)	61 (42.7)	74 (52.1)	33 (54.1)	261 (32.6)	
Female	270 (100.0)	92 (49.7)	82 (57.3)	68 (47.9)	28 (45.9)	540 (67.4)	
**Age group**							0.399
10–19	3 (1.1)	2 (1.1)	0 (0)	1 (0)	0 (0)	6 (0.7)	
20–29	2 (0.7)	7 (3.8)	1 (0.7)	1 (0.7)	2 (3.3)	13 (1.6)	
30–39	15 (5.6)	8 (4.3)	8 (5.6)	6 (4.2)	4 (6.6)	41 (5.1)	
40–49	26 (9.6)	26 (14.1)	18 (12.6)	7 (4.9)	4 (6.6)	81 (10.1)	
50–59	54 (20.0)	37 (20.0)	29 (20.3)	30 (21.1)	16 (26.2)	166 (20.7)	
60–69	60 (22.2)	49 (26.5)	37 (25.9)	44 (31.0)	18 (29.5)	208 (26.0)	
70–18	62 (23.0)	38 (20.5)	29 (20.3)	30 (21.1)	10 (16.4)	169 (21.1)	
80 +	48 (17.8)	18 (9.7)	21 (14.7)	23 (16.2)	7 (11.5)	117 (14.6)	
**Period of diagnosis**							0.093
2000–2003	39 (14.4)	38 (20.5)	21 (14.7)	29 (20.4)	7 (11.5)	134 (16.7)	
2004–2007	63 (23.3)	50 (27.0)	29 (20.3)	37 (26.1)	14 (23.0)	193 (24.1)	
2008–2011	92 (34.1)	69 (37.3)	59 (41.3)	44 (31.0)	21 (34.4)	285 (35.6)	
2012–2016	76 (28.1)	28 (15.1)	34 (23.8)	32 (22.5)	19 (31.1)	189 (23.6)	
**Total**	**270 (100.0)**	**185 (100.0)**	**143 (100.0)**	**142 (100.0)**	**61 (100.0)**	**801 (100.0)**	

The statistically significant values are highlighted in bold.

*Digestive organs, respiratory tract and intrathoracic organs, penis and other male urinary tract non-specified MM, genitourinary tract, peritoneum and retroperitoneum.

**There are no differences between male and female for sites such as vulva/vagina/cervix patients since they were removed from this test.

More than two thirds of patients had regional or distant metastases at the time of diagnosis. The majority of cases, ranging from 76.9% in anorectal MM to 89.2% in labial, pharynx and oral MM received any type of treatment. Treatment modalities were surgery combined with other modality at any point (65.3%), radiotherapy combined with other modality at any point (36.9%) and chemotherapy combined with other modality at any time (31.7%). Mortality for MM before the end of the first treatment or before 12 months in Brazil ranged from 17.1% in nasal and paranasal mucosa to 44.1% in digestive organs, respiratory tract and intrathoracic organs, penis and other male urinary tract non-specified MM, genitourinary tract, peritoneum and retroperitoneum MM. Regarding the status of patients at the end of first course of treatment, progressive disease, relapsed disease or death were the highest (68.9%) in anorectal MM and partial remission, stable disease and complete response reached 63.0% in nasal and paranasal MM (Table 3).

**Table 3:** Clinical characteristics of Mucosal Melanoma in Brazil.

Variables	Anatomic location					Total	p-value
	Vulva, vagina and uterine cervix	Lips, oral cavity and pharynx	Anal and rectal	Nasal and paranasal	Other mucosae*		
**Clinical stage (TNM)**							0.589
Localized Melanoma (Stages I and II)	40 (32.8)	16 (31.4)	12 (22.2)	7 (35.0)	4 (22.2)	79 (29.8)	
Regional or Distant metastases (Stages III and IV)	82 (67.2)	35 (68.6)	42 (77.8)	13 (65.0)	14 (77.8)	186 (70.2)	
Missing	122	134	148	89	43	536	
**Time from diagnosis to treatment**							0.500
≤60 days	90 (58.1)	67 (66.3)	48 (65.8)	48 (56.5)	15 (65.2)	268 (61.3)	
>60 days	65 (41.9)	34 (33.7)	25 (34.2)	37 (43.5)	8 (34.8)	169 (38.7)	
Missing	57	84	115	70	38	364	
**Specific treatment**							**0.027**
Yes	226 (83.7)	165 (89.2)	110 (76.9)	121 (85.2)	47 (77.0)	669 (83.5)	
No	44 (16.3)	20 (10.8)	33 (23.1)	21 (14.8)	14 (23.0)	132 (16.5)	
**Surgery**							** <0.001**
Yes	177 (78.3)	104 (63.0)	56 (50.9)	73 (60.3)	27 (57.4)	437 (65.3)	
No	49 (21.7)	61 (37.0)	54 (49.1)	48 (39.7)	20 (42.6)	232 (34.7)	
**Radiotherapy**							** <0.001**
Yes	56 (24.8)	74 (44.8)	40 (36.4)	67 (55.4)	10 (21.3)	247 (36.9)	
No	170 (75.2)	91 (55.2)	70 (63.6)	54 (44.6)	37 (78.7)	422 (63.1)	
**Chemotherapy**							**0.020**
Yes	61 (27.0)	49 (29.7)	49 (44.5)	36 (29.8)	17 (36.2)	212 (31.7)	
No	165 (73.0)	116 (70.3)	61 (55.5)	85 (70.2)	30 (63.8)	457 (68.3)	
Missing	21	20	44	33	14	132	
Response to the first course of treatment**							**0.009**
Response	59 (48.0)	46 (55.4)	19 (31.1)	34 (63.0)	12 (44.4)	170 (48.9)	
No response	64 (52.0)	37 (44.6)	42 (68.9)	20 (37.0)	15 (55.6)	178 (51.1)	
Missing	88	102	147	82	34	453	
Early death***							**0.025**
Yes	44 (29.5)	23 (24.0)	24 (35.3)	12 (17.1)	15 (44.1)	118 (28.3)	
No	105 (70.5)	73 (76.0)	44 (64.7)	58 (82.9)	19 (55.9)	299 (71.7)	
Missing	72	89	121	75	27	384	
**Total**	**270 (100.0)**	**185 (100.0)**	**143 (100.0)**	**142 (100.0)**	**61 (100.0)**	**801 (100.0)**	

*Digestive organs, respiratory tract and intrathoracic organs, penis and other male urinary tract non-specified MM, genitourinary tract, peritoneum and retroperitoneum.

**Response: partial remission, stable disease, and complete response; No response: progressive disease, relapsed disease or death.

***Death before the end of the first treatment or before 12 months.

The statistically significant values are highlighted in bold.

The univariate analysis of the risk factors for early death in mucosal melanoma is presented in Table 4, while the multivariate analysis is presented in Table 5. MM cases with regional or distant metastases as well as those located in the digestive organs, respiratory tract and intrathoracic organs, penis and other male urinary tract non-specified, genitourinary tract, peritoneum and retroperitoneum had almost 4 times more risk for early death. Besides that, MM located in lips, oral cavity and pharynx and those receiving chemotherapy had 2 times, anal and rectal MM had 2.6 times and MM in unusual locations had 3.8 times more risk of early death as opposed to nasal and paranasal MM. When the clinical stage was missing the risk of death was 3 times higher.

**Table 4:** Risk for early death* in Mucosal Melanoma in Brazil.

Variables	Early Death, N (%)		OR (95%CI)	p-value
	Yes	No		
**Anatomic location**				
Nasal and paranasal	12 (17.1)	58 (82.9)	Ref.	
Vulva, vagina and uterine cervix	44 (29.5)	105 (70.5)	1.5 (0.70–3.3)	0.290
Lips, oral cavity and pharynx	23 (24.0)	73 (76.0)	2.0 (1.0–4.14)	0.053
Anal and rectal	24 (35.3)	44 (64.7)	2.6 (1.2–5.8)	**0.017**
Other mucosae**	15 (44.1)	19 (55.9)	3.8 (1.5–9.6)	**0.004**
**Sex**				
Female	79 (27.7)	206 (72.3)	Ref.	
Male	39 (29.5)	93 (70.5)	1.1 (0.7–1.7)	0.700
**Age group**				
<60 years	42 (26.4)	117 (73.6)	Ref.	
≥60 years	76 (29.5)	182 (70.5)	1.2 (0.7–1.8)	0.503
**Períod of diagnosis**				
2000–2003	18 (30.5)	41 (69.5)	Ref.	
2004–2007	26 (26.8)	71 (73.2)	0.8 (0.4–1.7)	0.618
2008–2011	48 (31.0)	107 (69.0)	1.0 (0.5–2.0)	0.948
2012–2016	26 (24.5)	80 (75.5)	0.7 (0.4–1.5)	0.406
**Clinical stage (TNM)**				
Localized Melanoma (Stages I and II)	6 (11.5)	46 (88.5)	Ref.	
Regional or Distant metastases (Stages III and IV)	43 (36.1)	76 (63.9)	4.3 (1.7–11.0)	**0.002**
Missing	69 (28.0)	177 (72.0)	3.0 (1.2–7.3)	**0.017**
**Time from diagnosis to treatment**				
≤60 days	50 (33.6)	99 (66.4)	Ref.	
>60 days	21 (24.7)	64 (75.3)	0.7 (0.4–1.2)	0.158
Missing	47 (25.7)	136 (74.3)	0.7 (0.4–1.1)	0.117
**Surgery**				
Yes	65 (23.6)	210 (76.4)	Ref.	
No	53 (37.3)	89 (62.7)	1.9 (1.2–3.0)	**0.004**
**Radiotherapy**				
Yes	37 (24.5)	114 (75.5)	Ref.	
No	81 (30.5)	185 (69.5)	1.3 (0.9–2.1)	0.196
**Chemotherapy**				
No	64 (22.6)	219 (77.4)	Ref.	
Yes	54 (40.3)	80 (59.7)	2.3 (1.5–3.6)	**<0.001**
**Total**				

*Death before the end of the first treatment or before 12 months; Only treated patients were included in this analysis (N = 669).

**Digestive organs, respiratory tract and intrathoracic organs, penis and other male urinary tract non-specified MM, genitourinary tract, peritoneum and retroperitoneum.

**Table 5:** Independent risk factors for early death* in Mucosal Melanoma in Brazil.

Variables	^a^OR (95% CI)	p-value
Anatomic location: other mucosae**	3.8 (1.5–9.7)	0.005
Clinical stage (TNM): regional or distant metastases	3.6 (1.4–9.3)	0.008
Clinical stage (TNM): missing	3.1 (1.2–7.7)	0.015
Chemotherapy	2.1 (1.3–3.4)	0.002
Anatomic location: lips, oral cavity and pharynx	2.1 (1.0–4.5)	0.043

^a^OR = Adjusted odds ratio; CI = Confidence Interval.

*Death before the end of the first treatment or before 12 months; Only treated patients were included in this analysis (N = 669).

**Digestive organs, respiratory tract and intrathoracic organs, penis and other male urinary tract non-specified MM, genitourinary tract, peritoneum and retroperitoneum.

## Discussion

We believe that this is the largest scientific report of MMs considering its national perspective epidemiologically and the single one in the Americas. Two thirds of patients presented the disease at advanced stage with 28.3% of early death. A European study evaluated retrospectively the epidemiology of four hundred and forty-four individuals attending fifteen German skin cancer centers with MM. This German study reveal that anorectal, female genital tract and head and neck MMs prognostic differences are most likely related to early neoplastic events, before distant metastatic stage^6^, reinforcing the importance of early diagnosis also in grave and rare diseases such as MM. An Asian study evaluated the natural history and metastasis pattern prospectively for 706 patients with MM for 8 years^7^, although MM is the second most common type of melanoma in the continent, after acral lentiginous melanoma, very different from all other parts of the world. Few studies also evaluated MM from all mucosal surfaces, either from a single institution^8^ or from a national database^9^, although both presented a limited sample size. In addition, Schaefer presented 64% of death^8^ compared to 28.3% in our study, in 5 years and 1 year, respectively, which could explain such a difference, apart from the sample number limitations for a single institute of the earlier.

Aggressive tumors such as MM often have different genetic aberrations compared to cutaneous melanomas and are frequently treated with surgery^3^. Combined with other sort of treatment modality, in the present study surgery ranged from 50.9% in anorectal MM to 78.4% of female reproductive system in the sample studied (p < 0.001). With regards to Immunotherapy, which could minimize the surgical approach, such as involving anti-PD-1 agents, there is scientific evidence for use, not only in mucosal, but also in acral melanomas. Recently, response rates to PD-1 blockade in patients with acral and MMs were slightly lower but comparable to response rates in cutaneous melanomas and support the routine use of anti-PD-1 agents for MM^10^.

Staging of MM is essential, especially to determine if the disease reached distant metastasis stage^1,3,5,11^. Even though initial staging was not according to the new staging classification for MM^12^, it is clear most cases were diagnosed already with distant metastases in Brazilian patients, highlighting the relevance for an early diagnosis of the disease. In addition, for head and neck mucosal melanoma (HNMM), primary tumor size seems to be associated with distant metastasis^13^.

In Brazil the 60 days law determines that the public health treatment for any malignancy should start within this time frame^14^. Time between diagnosis and treatment could be implicated as critical to MM patients from Brazil. Over a third of patients (38.7%) had to wait more than 60 days from diagnosis to treatment. Most patients with MM in unusual locations (digestive organs, respiratory tract and intrathoracic organs, penis and other male urinary tract non-specified, genitourinary, peritoneum and retroperitoneum) showed up at the hospital without diagnosis or previous treatment, which probably means that required expertise and technology for diagnosis were only available at the cancer hospitals network and not at primary or secondary care. The dramatic therapeutic advances in melanoma field and the poor prognosis of patients with MM mandates continued emphasis on laboratory and clinical research efforts in this rare and serious disease^11^ not only in Brazil, but worldwide.

Stage IV was the clinical initial staging for most cases in this study (ranging from 36.6% in vulva, vagina and cervix to 66.7% for other mucosae). It is recommended to suppress T1 and T2 skipping the primary tumor from T0, given that MM are aggressive tumors. When the primary tumor is not detectable or it is superficially located, it should be staged as T3. Moderate or very advanced disease should be staged as T4^12^ The AJCC published further data regarding melanoma staging^15^, nonetheless, MM is not mentioned specifically^16^.

A specific TNM classification was developed for skin and ocular lesions and a very basic classification for HNMM which was designed as follows: stage I when only local disease is present; stage II, when neck lymph node metastasis is present and stage III: when distant metastasis is detected^5^. Nevertheless, upper aerodigestive tract MM are present at the new TNM classification. This classification is only directed to HNMM. Although, Ballester Sánchez *et al*. takes into account the Ballantyne simplified staging method to all MM, with the single modification related to stage II in neck lymph nodes, which extrapolates to the regional lymph node involvement^1^. In order to provide an ideal treatment planning and prognostication, there is a need for the validation of specific staging methods for all locations of MMs^2^.

Some limitations of this study are related to the fact that it is based on secondary database. Hence, there was no histopathological, immunological or molecular revision of MM, also making it difficult to discuss further therapeutic opportunities in detail. In addition, significant variables such as C51.0 category (labium majus) from ICD-O-3 does not allow to distinguish between skin and mucosa, making it possible that some of the vulvar melanoma could be from the skin instead of mucosa. Ulceration, thickness or depth of the primary tumor for localized disease, number of metastatic lymph node for regional disease, and the metastatic organ would be better predictors of prognosis. Also, the survival analysis with identification of associated factors by Cox regression would be more appropriate, but available data do not include time between diagnosis and death or last follow-up and it was not possible to perform Kaplan Meier/Cox Regression analyzes since this is a public domain secondary database analysis with pre-defined variables. Last but not least, the absence of important variables such as histopathological, molecular, clinical and therapeutic variables, as well as date of diagnosis and date of death were mostly not available. Strengths include a large sample size, all anatomic sites presenting with MM and evaluation of the disease in a real-life setting.

Late diagnosis and early death are eminent challenges to the Brazilian healthcare system related to MM management. A recently published whole genome sequencing study revealed a heterogeneous MM profile based not only on the pattern of mutations but also on body site-specific driver mutations with genetic ancestry or geographic location playing a significant role^17^. Nevertheless, such studies need to be conducted with larger cohorts and preferably with all sorts of geographic locations, including the genetically diverse Brazilian population. It may safely be said, personalized medicine for MM is on the way.

The insertion of mutational profile of MMs should be always considered in routine clinical settings in order to evaluate the applicability of the available target therapy for each case. It appears that the mutational landscape of mucosal melanoma points out to a distinct pattern between the upper and lower regions of MM commitment with SF3B1 and KIT presenting higher mutation rates than the common drivers of cutaneous melanomas, namely BRAF and NRAS. This slightly different mutational profile amongst MMs might help provide new comprehension of this grave disease and personalize therapeutic options for such patients in the era of precision medicine^18^. In addition, MMs usually present a low point mutation burden even though with a high number of structural variants. SF3B1, KIT, BRAF, NRAS, NF1, TP53, SPRED1, CHD8, ATRX, and HLA-A are the genes that usually present significant mutations. Yet, in terms of structural variants, TERT, MDM2 and CDK4 are the most usual targets to present structural rearrangements^17^.

## Methods

### Study population

Study population. A retrospective cohort study was conducted with patients diagnosed with MM between 2000 and 2016. Information from Brazilian hospital-based cancer registries, obtained through the Integrator System (Brazilian National Cancer Institute – INCA, available at https://irhc.inca.gov.br/RHCNet/visualizaTabNetExterno.action) and São Paulo’s Hospital Cancer Registry (Oncocentro Foundation, available at http://200.144.1.68/cgi-bin/dh?rhc/rhc-geral.def) were merged. The final database included information from 310 cancer hospitals from the 25 states and the Federal District of Brazil. Data were obtained on August 7, 2018. There was no central pathology review.

### Data analysis

Patients were followed up until the end of first course of treatment (INCA) or 12 months (Oncocentro Foundation). The following variables were collected: age at diagnosis (in years), sex, year of diagnosis, anatomic location and histological type (according to the International Classification of Diseases for Oncology, third Edition - ICD-O-3), clinical stage at diagnosis (Localized Melanoma - Stages I and II versus Regional or Distant metastases - Stages III, with lymph node metastasis and IV, with distant metastasis), time from diagnosis to treatment (≤60 days versus >60 days), first-course therapy (surgery, chemotherapy, radiotherapy) and status at the end of the first course of treatment, classified using Surveillance, Epidemiology and End Results - SEER - definitions^19^. The considered outcome was early death, defined as death before the end of the first treatment or before 12 months.

### Statistical analysis

A descriptive analysis was performed using frequencies for the categorical variables. Missing values have been excluded from analysis and only valid percents were shown. The χ2 test was used to compare categorical variables. A univariate analysis was performed using Odd Ratios (OR) with 95% confidence intervals (95% CI). Forward stepwise logistic regression method was used in the multivariate analysis to identify independent predictors of early death. Differences were considered statistically significant when p values were <0.05. The statistical software package used was SPSS, version 21.0 (São Paulo, Brazil).

### Ethical approval

The Ethics in Human Research Committee of the Brazilian National Cancer Institute (CEP-INCA) approved this study in Rio de Janeiro, Brazil (ref. number 128/11 CAAE – 0104.0.007.000-11) on September, 26^th^, 2011. All research was performed in accordance with relevant guidelines and regulations from CEP-INCA. Informed consent for this type of study is not required because it is based on secondary publicly available database from Brazil, which was confirmed via the ethics committee opinion 128/11, after the study acceptance.

## Data Availability

The authors make materials and data from the present article promptly available to readers without undue qualifications in material transfer agreements. There is no restriction on the availability of materials or information.
